# Knockdown of long noncoding RNA metastasis-associated lung adenocarcinoma transcript 1 protects against intracerebral hemorrhage through microRNA-146a-mediated inhibition of inflammation and oxidative stress

**DOI:** 10.1080/21655979.2022.2031401

**Published:** 2022-02-03

**Authors:** Zhanyi Xu, Baoshuai Zhao, Jianhui Mao, Zhaosheng Sun

**Affiliations:** Department of Neurosurgery, Hengshui People’s Hospital, Hengshui China

**Keywords:** Intracerebral hemorrhage, lncRNA MALAT1, miR-146a, inflammation, oxidative stress

## Abstract

Studies have demonstrated that long noncoding RNAs (lncRNAs) are important regulators of intracerebral hemorrhage (ICH) and participants in ICH pathogenesis. We designed this study to probe the potential functions and mechanisms of lncRNA metastasis-associated lung adenocarcinoma transcript 1 (MALAT1) in ICH. The ICH model was established and the rats were treated with MALAT1-shRNA or MALAT1-shRNA+miR-146a inhibitor 1 h after ICH induction. A dual-luciferase reporter assay was employed to examine the relationship between MALAT1 and miR-146a. In addition, rat neurobehavioral changes, brain water content, and neuronal apoptosis were measured in this study. Furthermore, the pro‑inflammatory markers tumor necrosis factor alpha (TNF-α) and interleukin (IL)-1β were determined by enzyme-linked immunosorbent assays (ELISAs), while the oxidative stress factors, including malondialdehyde (MDA) and superoxide dismutase (SOD), were also evaluated. Lastly, a Western blot assay was employed to examine the protein levels of phosphorylated (p)-p65 and p65. First, we found that MALAT1 was expressed at higher levels in ICH rats. miR-146a is a target gene of MALAT1 and is downregulated in ICH rats. Downregulation of MALAT1 inhibited the neurological scores, brain water content, and neuronal apoptosis, reduced the levels of pro-inflammatory cytokines, and prevented oxidative stress in ICH rats. In addition, the protein level of p-p65 and the ratio of p-p65/p65 were decreased in the MALAT1-shRNA group. All the effects of MALAT1-shRNA on ICH rats were reversed by miR-146a inhibitor co-treatment. In conclusion, downregulation of MALAT1 protected against ICH by suppressing inflammation and oxidative stress by upregulating miR-146a.

## Graphical Abstract

**Figure:**
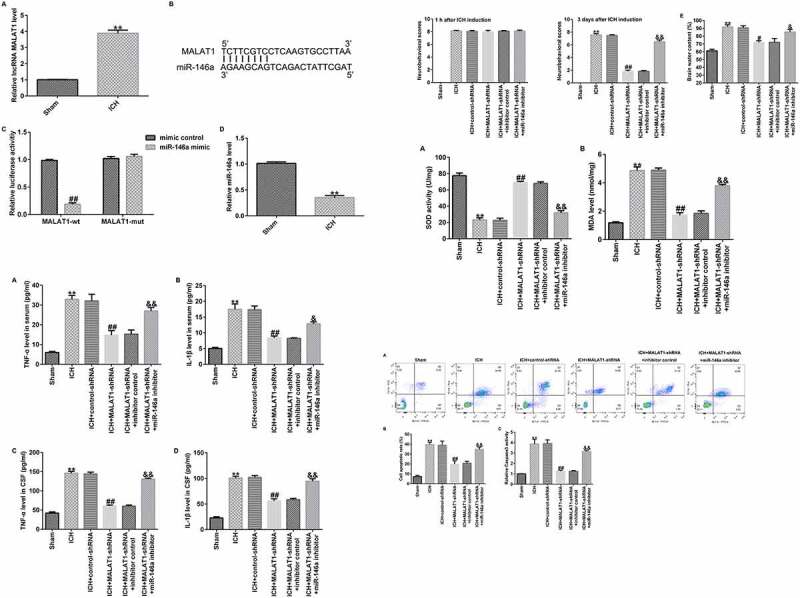

## Introduction

Intracerebral hemorrhage (ICH) is a common acute neurological disorder that accounts for approximately 15% of strokes and carries a high risk of morbidity and disability [1,2]. At present, effective therapeutic options for ICH are limited, and few pharmacological or surgical therapies provide great benefits [3]. ICH injury can be classified into two continuous but indivisible processes, namely primary brain damage and secondary brain damage [4]. Primary brain injury is the initiation and dilation of the hematoma, resulting in physical injury to brain tissues [5]. Primary brain damage then exacerbates various pathological processes, such as oxidative stress, neuronal death (including apoptosis and necrosis), inflammation, and reactive oxygen species (ROS) generation, resulting in secondary brain injury (SBI) [6]. Thus, probing the regulatory mechanism of SBI may be a promising policy for the treatment of ICH.

Long noncoding RNA (lncRNA), a type of non-coding RNA consisting of more than 200 nucleotides, has no translational activity [7]. Multiple studies have shown that lncRNAs participate in diverse biological processes, such as cell proliferation, apoptosis, tumorigenesis, and metastasis [8,9]. In addition, changes in lncRNA may result in various diseases, including neoplastic disorders, dementia, epilepsy, and ICH [10–13]. It has been reported that the lncRNA NKILA induces the endoplasmic reticulum stress/autophagy pathway and inhibits the nuclear factor-k-gene binding pathway in ICH rats [14]. Zhang et al. demonstrated that lncRNA Snhg3 resulted in the dysfunction of cerebral microvascular cells in ICH rats by activating the TWEAK/Fn14/STAT3 pathway [15]. Metastasis-associated lung adenocarcinoma transcript 1 (MALAT1) is a highly conserved nuclear lncRNA that was initially identified as a premonitory symbol for lung cancer metastasis [16]. An increasing number of studies have illustrated the important roles of MALAT1 in the progression of diseases associated with inflammation, apoptosis, and oxidative stress [17–19]. Fang et al reported that MALAT1 silencing enhances the cerebral protection of dexmedetomidine against hypoxic-ischemic brain damage through the inhibition of apoptosis of hippocampal neurons by suppressing Wnt family member 1 expression [17]. MALAT1 promotes inflammatory responses in Parkinson’s disease [18]. Moreover, MALAT1 plays an important role in cardiovascular disease by controlling oxidative stress [19]. These findings suggested that MALAT1 plays key roles in the regulation of inflammation, apoptosis, and oxidative stress which are key factors in SBI. To date, the function of MALAT1 in ICH remains unclear. We hypothesized that MALAT1 plays a role in ICH through regulating neuronal inflammation, apoptosis, and oxidative stress.

MicroRNAs (miRNAs), a series of short single-stranded ncRNAs, are known to regulate various biological processes, including organ development, proliferation, inflammation, and tumorigenesis [20]. Recent research has indicated that the expression of miRNAs plays essential roles in regulating human diseases, including ICH [21–23]. MiR-146a, located on chromosome 5, may regulate many illnesses such as spinal cord injury, myocardial dysfunction, inflammation, and ICH [24,25]. Qu et al. demonstrated that miR-146a could prevent ICH by suppressing inflammation and oxidative stress [25]. Additionally, it is widely known that miRNAs can interact with lncRNAs to determine their biological functions. Dai et al. showed that downregulation of lncRNA MALAT1 inhibited the inflammatory response by upregulating miR-146a in LPS-induced acute lung injury [26]. However, whether lncRNA MALAT1 could exert effects on ICH by targeting miR-146a remains to be explored.

In this study, we hypothesized that MALAT1 plays an important role in ICH through regulating neuronal inflammation, apoptosis, and oxidative stress via miR-146a. Therefore, this study was designed to to assess the functions of MALAT1 and the underlying molecular mechanisms of ICH. Furthermore, whether miR-146a interacts with the effects of MALAT1 was also addressed.

## Materials and methods

### Animals

Adult male Sprague–Dawley rats weighing 280–320 g were acquired from the Animal Center of the Chinese Academy of Sciences (Shanghai, China). The rats were kept in a humidity-and temperature-controlled environment and a 12 h/12 h light-dark cycle, with adequate food and water. All animal procedures were performed in accordance with the National Institutes of Health guidelines and were approved by the Animal Care and Use Committee of Hengshui People’s Hospital (No. AF/SC-08/02.0).

### Cell culture

HEK293 cells were obtained from the American Type Culture Collection (ATCC, Manassas, VA, USA). RPMI 1640 medium supplemented with 10% FBS and 1% P/S was used to culture cells, which were then maintained at 37°C in a 5% CO_2_ incubator.

### Dual luciferase reporter assay [27]

Studies have reported that there is a binding site between MALAT1 and miR-146a. To investigate direct target binding, WT and MUT of lncRNA MALAT1 3′-UTRs were inserted into a pmirGLO vector. HEK293 cells were plated into 24-well plates and transfected with WT‑MALAT1 or MUT‑MALAT1 and mimic control or miR-146a mimic using Lipofectamine 3000 reagent (Invitrogen, Waltham, MA, USA). The luciferase activity of HEK293 cells was determined using a dual-luciferase assay system (Promega, Madison, WI, USA). Luciferase activity was standardized relative to *Renilla* luciferase activity.

### Establishment of intracerebral hemorrhage model [25]

To establish the ICH model, rats were anesthetized with 5% chloralhydrate (250 mg/kg) by intraperitoneal injection [25]; no rats exhibited signs of peritonitis, pain, or discomfort. After anesthetization, collagenase type VII (Sigma-Aldrich, St. Louis, MO, USA) was slowly injected into the left striatum through stereotactic infusion. After infusion, the needles were kept at the injection site for 5 min. Then, the needle was slowly dismantled, and the burr hole was sealed with bone wax. Sham rats were infused with an equal amount of saline buffer. The control-shRNA (2 μg/μl), ALAT1-shRNA (2 μg/μl), MALAT1-shRNA (2 μg/μl)+inhibitor control (2 μg/μl), or MALAT1-shRNA (2 μg/μl)+miR-146a inhibitor (2 μg/μl) were added to Entranster™ in vivo transfection reagent (1.25 μl) and the solution was mixed gently and left for 15 min. After induction of ICH for 1 h, the solution was intracerebroventricularly injected into rats as described previously [28]. A total of 60 rats were randomly divided into following six groups (n = 10): Sham, ICH, ICH+control-shRNA, ICH+MALAT1-shRNA, ICH+MALAT1-shRNA+inhibitor control, and ICH+MALAT1-shRNA+miR-146a inhibitor group. We did our best to alleviate the pain in rats, and the experiments were terminated when the rats lost >15% of their body weight.

### Neurological score evaluation [29]

Neurobehavioral tests were performed 1 h and 3 d post-ICH induction or sham surgery in all rats using the modified neurological severity score method (mNSS). The mNSS tests consisted of motion tests, sensory tests, beam balance tests, and a lack of reflection and abnormal motion. The neurological function score was 0‑18 (normal neurological score, 0; maximal deficit score, 18).

### Brain edema assessment [25]

To evaluate brain edema in different groups, we confirmed the brain water content. In brief, rats were anesthetized using intraperitoneal 4% chloral hydrate and then beheaded. Then, brain tissues were separated into two hemispheres, and the hemispheres were further divided into two sections, including the cortex and basal ganglia. After removing the hematoma, the tissues were weighed to obtain the wet weight. The dry weight was measured after baking in an oven at 160°C for 24 h. Lastly, the formula: [(wet weight − dry weight)/wet weight]×100% was used to count the brain water content.

### Flow cytometric analysis [30]

To assess brain cell apoptosis, the Annexin V/propidium iodide (PI) Cell Apoptosis Detection Kit (KeyGen Biotech, Nanjing, China) was used. Brain tissue was isolated and then separated into cell suspensions using a neural tissue dissociation kit (Miltenyi Biotec, Bergisch Gladbach, Germany). Furthermore, cells were re-suspended in binding buffer, 5 μl of annexin V, and 5 μl of PI, and incubated for 15 min while avoiding light. Finally, the cells were analyzed via flow cytometry using FlowJo software (version 10.0).

### ELISA [31]

After ICH induction, blood and cerebrospinal fluid (CSF) were collected after puncture of the heart and macropore. Then, the CSF and blood samples from rats were centrifuged at 12,000 × g for 30 min and 1,000 × g for 5 min at 4°C. The concentrations of TNF-α and IL-1β in the supernatants were detected using an ELISA kit (Beyotime, Shanghai, China).

### Detection of oxidative stress factors [25]

The thiobarbituric acid reaction method was employed to determine malondialdehyde (MDA) levels. First, 100 μl homogenate samples and 100 μl SDS cracking buffer were added to the microcentrifuge tube, shaken fully, and cultured at room temperature for 5 min. In addition, 250 μl thiobarbituric acid was added and the cells were cultured at 95°C for 1 h, followed by centrifugation at 3000 rpm for 15 min. After that, 300 μl supernatant was added to another tube containing 300 μl n-butanol. Finally, the tube was centrifuged at 10,000 *g* at 4°C for 5 min, and the absorbance was measured at 532 nm on a microplate reader.

An SOD assay kit (Beyotime, Haimen, China) was used for the detection of superoxide dismutase (SOD) activity. The brain tissues were homogenized and centrifuged at 12,000 × g for 5 min at 4°C. Superabundant cells were used for the SOD assay following the manufacturer’s protocol. Finally, the OD_450_ was measured using a microplate reader (BioTek, USA).

### Reverse transcriptase quantitative polymerase chain reaction (RT-qPCR)

TRIzol reagent (Invitrogen) was used to isolate total RNA from the brain tissues. Total RNA was then transcribed into cDNA using the QuantiTect Reverse Transcription Kit (Qiagen, Hilden, Germany). The quantitative real-time (qRT)-PCR assay was performed using a Rotor-Gene SYBR Green PCR Kit with the following reaction: 5 s at 95°C and 10s at 60°C for 45 cycles after an initial denaturation step of 5 min at 95°C. RNA abundance was determined using the 2^−ΔΔCq^ method [32], U6 or GAPDH was adopted as the internal control.

### Western blot assay [33]

Briefly, the brain tissues were collected, and the total protein was extracted using RIPA lysate buffer (Beyotime). Protein concentrations were determined using the BCA kit (Beyotime). Then, equal amounts of samples were applied to 10–12% SDS-polyacrylamide gel, separated, and then transferred to PVDF membranes. In addition, the membranes were blocked in 5% skim milk for 60 min at RT and incubated with the following primary antibodies: p-p65 (cat. no. ab76302; Abcam, Cambridge, UK; 1:1000), p65 (cat. no. ab19870; Abcam; 1:1000), and GAPDH (cat. no. ab22555; Abcam; 1:1000) overnight at 4°C. After that, the membranes were incubated with an HRP-conjugated secondary antibody (cat. no. ab96899; Abcam, 1:2000) at RT for 1 h. Finally, the protein bands were visualized using enhanced chemiluminescence (ECL).

### Statistical analysis

Each measurement was processed using SPSS (version 20.0; SPSS, Chicago, IL, USA) and expressed as means ± SD. One-way ANOVA with a Bonferroni post hoc test was employed for statistical analysis of all data between multiple groups. Student’s *t*-test was used to determine the significance of differences between two independent groups. Significant differences between the groups were set at p < 0.05.

## Results

### Long noncoding RNA MALAT1 was upregulated in intracerebral hemorrhage rats

**Figure 1.**
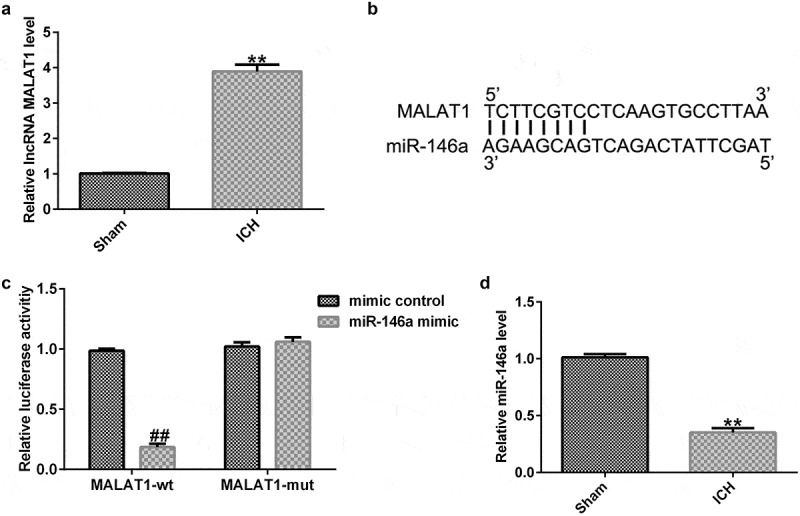
Long noncoding RNA MALAT1 was upregulated in intracerebral hemorrhage rats targeted miR-146a and miR-146a was downregulated. (a)The expression levels were detected using quantitative real-time PCR (qRT-PCR). (b) Binding sites between MALAT1 and miR-146a. (c) Dual-luciferase analysis proved that the miR-146a mimic repressed the luciferase activity of WT‑MALAT1, while no obvious effects on MUT‑MALAT1 activity. (d) The qRT-PCR assay was used to check miR-146a levels in intracerebral hemorrhage rats. Results are displayed as mean ± standard deviation, **p < 0.01, vs. sham; ##p < 0.01 vs. mimic control.

To determine the function of lncRNA MALAT1 in ICH, an *in vivo* ICH model was conducted, and qRT-PCR assay was then performed to check the MALAT1 levels in ICH and sham rats. As presented in Figure 1a, the MALAT1 level was higher in ICH rats than in sham rats.

### MALAT1 targeted miR-146a and miR-146a was downregulated in ICH rats

Previous studies have shown the binding sites between MALAT1 and miR-146a (Figure 1b). In the present study, a luciferase reporter assay was performed to determine the binding region. Briefly, HEK293 cells were co-transfected with the pmirGLO vector containing the 3′-UTR of MALAT1 together with miR-146a mimic or mimic control. As demonstrated in Figure 1c, the luciferase activity of WT‑MALAT1 was markedly decreased in the miR-146a mimic group compared to the mimic control, while no obvious promotion in MUT‑MALAT1 activity was observed.

Moreover, qRT-PCR was performed to determine miR-146a levels in ICH and sham rats. In the ICH group, the level of miR-146a was lower than that in the sham group (Figure 1d). These data showed that MALAT1 directly targeted miR-146a, and miR-146a was decreased in ICH rats.

### Downregulation of MALAT1 regulated miR-146a expression in ICH rats

To determine the effects of MALAT1 in ICH rats, after ICH induction for 1 h, control-shRNA, MALAT1-shRNA, MALAT1-shRNA+inhibitor control, or MALAT1-shRNA+miR-146a inhibitor were transfected into ICH rats via intraventricular injection.

**Figure 2.**
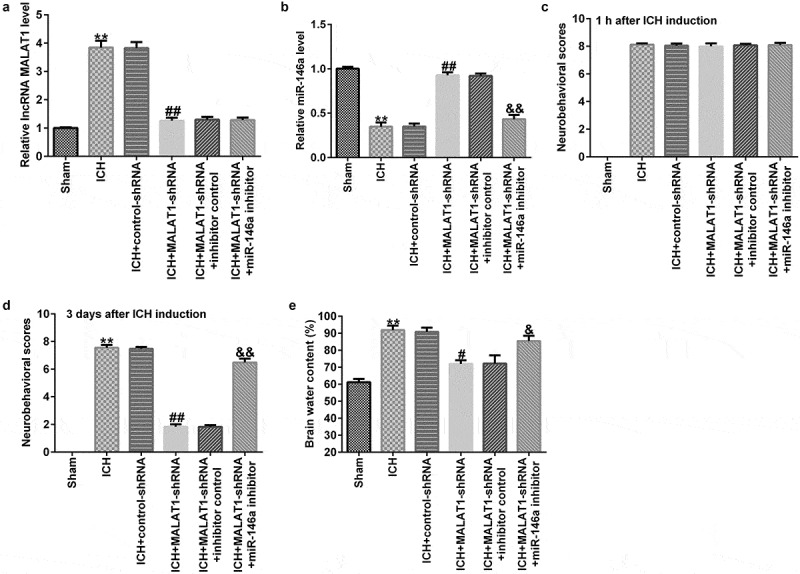
Inhibition of long noncoding RNA MALAT1 reduced neurological damage and brain edema in intracerebral hemorrhage rats. After 1 h of intracerebral hemorrhage (ICH) induction, control-shRNA, lncRNA MALAT1-shRNA, MALAT1-shRNA+inhibitor control, or MALAT1-shRNA+miR-146a inhibitor were transfected into ICH rats. (a) MALAT1 levels in different groups. (b) Expression of miR-146a in different groups. Neurobehavioral scores were assessed using the mNSS test in various groups after ICH for 1 h (c) and 3 d (d). (e) Brain water content was investigated using the wet/dry method in all groups. Data are displayed as means ± standard deviation, **p < 0.01 vs. sham; #, ##p < 0.05, 0.001 vs. ICH+control-shRNA; &, &&p < 0.05, 0.01 vs. ICH+MALAT1-shRNA+inhibitor control.

To probe the correlation between MALAT1 and miR-146a in ICH rats, qRT-PCR was performed to verify the transfection efficiency. As shown in Figure 2a, the expression of MALAT1 in the ICH group was higher than that in the sham group, and the expression of MALAT1 was reduced by MALAT1-shRNA in ICH rats compared to ICH + control-shRNA rats. In addition, compared to the sham rats, the expression of miR-146a was decreased in ICH rats. The expression of miR-146a was higher in the MALAT1-shRNA-transfected ICH rats than in the ICH + control-shRNA rats. Furthermore, when compared to the MALAT1-shRNA+inhibitor control-transfected ICH rats, the expression of miR-146a was decreased in MALAT1-shRNA+miR-146a inhibitor co-transfected ICH rats (Figure 2b).

### Downregulation of MALAT1 reduced neurological damage

To assess the neurological damage of the rats, neurobehavioral scores were assessed at 1 h and 72 h after ICH induction or sham operation in all groups using the mNSS test. Our results demonstrated that after ICH induction for 1 h, the neurobehavioral score in ICH rats was higher than that in sham rats, while no obvious differences were observed among the other groups (Figure 2c). After ICH induction for 72 h, the neurobehavioral score in ICH rats was also higher than that in sham rats, and the neurological score in the MALAT1-shRNA group was lower than that in the ICH + control-shRNA group. In addition, compared with the ICH + MALAT1-shRNA+inhibitor control group, the neurological score was evidently higher in the ICH + MALAT1-shRNA+miR-146a inhibitor group in ICH rats (Figure 2d). These results indicated that downregulated MALAT1-shRNA could reduce neurological damage in ICH rats, and the effects were reversed by the miR-146a inhibitor.

### Downregulation of MALAT1 decreased brain edema

Since brain edema is a necessary part of ICH-associated brain injury, the brain water content was detected via the wet/dry method. The results suggested that brain water content was notably higher in ICH rats than in sham rats. In addition, compared to the ICH group, the brain water content was notably reduced in the MALAT1-shRNA group. Brain water content in the MALAT1-shRNA+miR-146a inhibitor group was enhanced further compared with that in the MALAT1-shRNA group in ICH rats (Figure 2e).

### Downregulation of MALAT1 suppressed pro‑inflammatory cytokine levels

**Figure 3.**
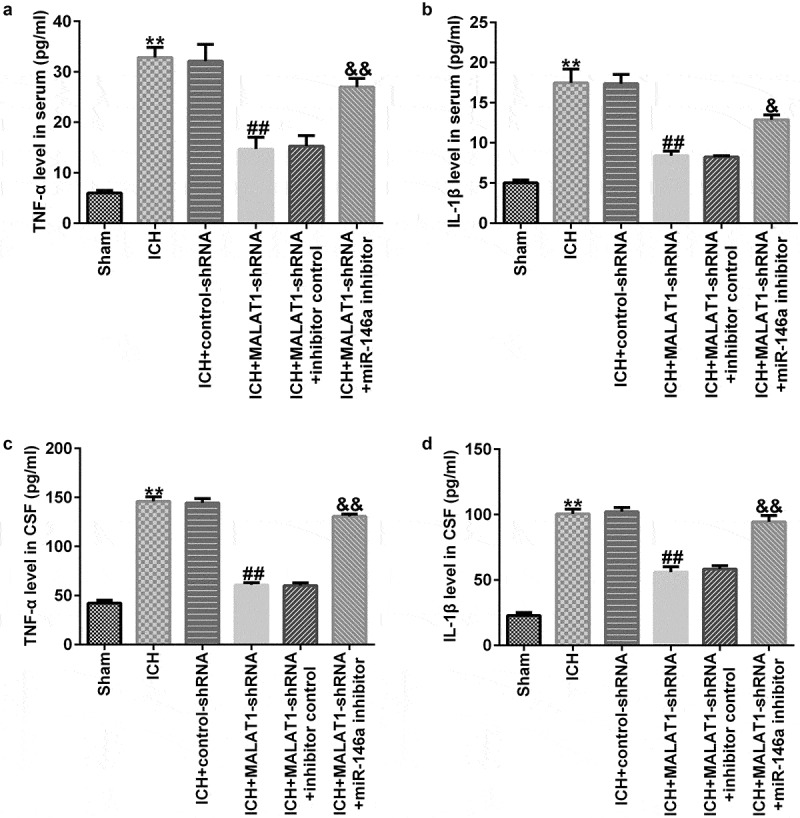
Downregulation of long noncoding RNA MALAT1 inhibited the pro‑inflammatory cytokine levels in intracerebral hemorrhage rats. ELISA was employed to determine the TNF-α and IL-1β levels in the serum and cerebrospinal fluid in rats of sham, ICH, ICH+control-shRNA, ICH+MALAT1-shRNA, ICH+MALAT1-shRNA+inhibitor control, and ICH+MALAT1-shRNA+miR-146a inhibitor groups. Data are expressed as the mean ± SD. **p < 0.01 vs. Sham; ##p < 0.001 vs. ICH+control-shRNA; &, &&p < 0.05, 0.01 vs. ICH+MALAT1-shRNA+inhibitor control.

Next, we explored whether MALAT1 exerted anti-inflammatory effects on ICH. The data indicated that in comparison to the sham group, the pro‑inflammatory markers TNF-α and IL-1β in the serum and CSF were obviously upregulated after ICH. The levels of TNF-α and IL-1β were lower in the MALAT1-shRNA group than in the ICH + control-shRNA group. In addition, in comparison with MALAT1-shRNA+inhibitor control in ICH, the levels of TNF-α and IL-1β in the serum and CSF of ICH rats in the MALAT1-shRNA+miR-146a inhibitor group were significantly enhanced (Figure 3).

### Downregulation of MALAT1 inhibited oxidative stress

**Figure 4.**
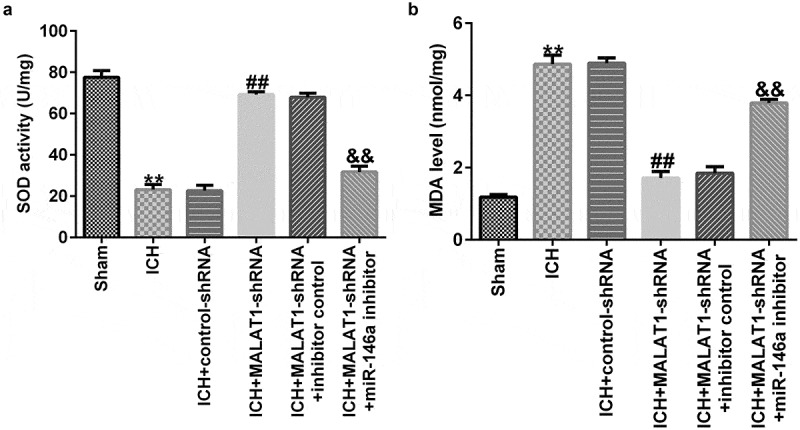
Downregulation of long noncoding RNA MALAT1 suppressed oxidative stress in intracerebral hemorrhage rats.

Exaggerated oxidative stress results in ICH-induced brain damage; thus, levels of oxidative stress biomarkers, such as MDA and SOD, were determined. As illustrated in Figure 4, SOD activity was lower and MDA levels were markedly enhanced in ICH rats compared to those in the sham group. The results also indicated that downregulated MALAT1 notably promoted SOD activity and suppressed the level of MDA compared to the ICH group. Moreover, SOD activity was inhibited and MDA levels were upregulated in the MALAT1-shRNA+miR-146a inhibitor group compared to the MALAT1-shRNA+inhibitor control group in ICH rats.

SOD activity and MDA levels were measured in rat brain tissues from different groups. Results are displayed as mean ± SD. **p < 0.01 vs. Sham; ##p < 0.001 vs. ICH+control-shRNA; &&p < 0.01 vs. ICH+MALAT1-shRNA+inhibitor control.

### Downregulation of MALAT1 inhibited neuronal apoptosis

**Figure 5.**
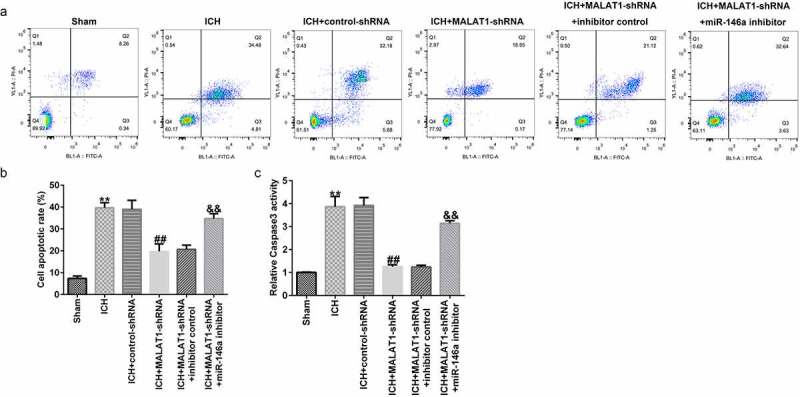
Inhibition of long noncoding RNA MALAT1 inhibited neuronal apoptosis in intracerebral hemorrhage rats.

ICH-induced neuronal apoptosis plays an essential role in secondary brain damage. Thus, neuronal apoptosis and caspase3 activity were judged to determine the effects of MALAT1 on neurons. In comparison to the sham group, apoptotic cells and caspase3 activity were distinctly upregulated in the ICH group. In addition, the apoptotic cells and caspase3 activity were lower in the MALAT1-shRNA group than in the ICH + control-shRNA group. Moreover, in comparison with the MALAT1-shRNA+inhibitor control group, the apoptotic cells and caspase3 activity were increased in the MALAT1-shRNA+miR-146a inhibitor group (Figure 5a–c). The above data indicated that MALAT1 participates in neuronal apoptosis after ICH.

(A and B) The neuronal apoptosis was detected using flow cytometric analysis in the brain tissue of rats from sham, ICH, ICH + control-shRNA, ICH + MALAT1-shRNA, ICH + MALAT1-shRNA+inhibitor control, and ICH + MALAT1-shRNA+miR-146a inhibitor groups. (C) Caspase3 activity in the brain tissue of rats from sham, ICH, ICH + control-shRNA, ICH + MALAT1-shRNA, ICH + MALAT1-shRNA+inhibitor control, and ICH + MALAT1-shRNA+miR-146a inhibitor groups was calculated. Results are expressed as mean ± SD. **p < 0.01 vs. sham; ##p < 0.001 vs. ICH + control-shRNA; &&p < 0.01 vs. ICH + MALAT1-shRNA+inhibitor control.

### Downregulation of MALAT1 inhibited the NF‑κB pathway

**Figure 6.**
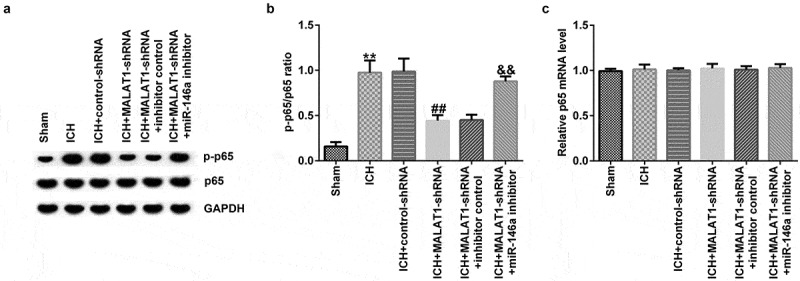
Downregulation of long noncoding RNA MALAT1 suppressed the NF‑κB pathway in intracerebral hemorrhage rats.

To further elucidate the molecular mechanism by which MALAT1 plays a protective role in ICH, we investigated whether the NF-κB pathway was involved. It was demonstrated that the protein level of p-p65 and the ratio of p-p65/p65 were notably upregulated in ICH rats compared to the sham rats. We also found that the level of p-p65 and the ratio of p-p65/p65 were decreased in the MALAT1-shRNA group compared to the ICH + control-shRNA group. In addition, in comparison to the MALAT1-shRNA+inhibitor control group, the level of p-p65 and the ratio of p-p65/p65 were enhanced in the MALAT1-shRNA+miR-146a inhibitor group in ICH rats (Figure 6a and b). The mRNA levels of p65 showed no significant changes between the groups (Figure 6c).

Western blot assay was performed to determine the protein expression of p-p65 (A) and the ratio of p-p65/p65 (B) in the brain tissue of rats from the sham, ICH, ICH + control-shRNA, ICH + MALAT1-shRNA, ICH + MALAT1-shRNA+inhibitor control, and ICH + MALAT1-shRNA+miR-146a inhibitor groups. (C) The mRNA levels of p65 were determined using qRT-PCR. Results are shown as mean ± SD. **p < 0.01 vs. sham; ##p < 0.001 vs. ICH + control-shRNA; &&p < 0.01 vs. ICH + MALAT1-shRNA+inhibitor control.

## Discussion

ICH is the most severe type of stroke, causing high mortality and disability [1]. Accumulating evidence has shown that lncRNAs play an essential role in many illnesses, such as neoplastic disorders, dementia, epilepsy, and ICH [11,13]. However, the involvement of the MALAT1 in ICH has not been investigated. The current study is the first to prove that MALAT1 is upregulated in ICH rats. In addition, we disclosed new functions and mechanisms of MALAT1 in ICH and demonstrated that MALAT1 downregulation protected against ICH via the suppression of inflammatory response and oxidative stress through upregulation of miR-146a.

MALAT1, a crucial lncRNA, is expressed in many tissue types. It participates in various diseases and biological processes. In the current study, we noticed that the MALAT1 level was higher in ICH rats than in the sham group. MiR-146a is considered to be associated with the progression of many illnesses, including spinal cord injury, myocardial dysfunction, and ICH [17]. Recently, several study has shown that miR-146a is a direct target of MALAT1 [26]. In accordance with previous study, results from a dual-luciferase assay proved that MALAT1-shRNA promoted WT‑miR-146a activity. In addition, miR-146a levels were lower in ICH rats than in sham rats.

To further explore the effects of MALAT1 and miR-146a in ICH, control-shRNA, MALAT1-shRNA, MALAT1-shRNA+inhibitor control, or MALAT1-shRNA+miR-146a inhibitor were transfected into ICH rats via intraventricular injection. The findings indicated that MALAT1 negatively regulated the miR-146a expression in ICH rats. Neurological scores and brain edema are the most important markers of ICH [34]. In the present study, we found that MALAT1-shRNA reduced the neurological score and brain water content in comparison to the ICH group. However, the results were reversed in the MALAT1-shRNA+miR-146a inhibitor group. However, this research did not assess the hematoma volume in rats and did not perform immunohistochemistry on rat brain tissue, which was a limitation of this study. Neuronal apoptosis, inflammatory response, and oxidative stress are the three key factors in SBI [35–37]. Results from the current study showed that cell apoptosis and oxidative stress were reduced in the MALAT1-shRNA group. The results were also reversed in the MALAT1-shRNA+miR-146a inhibitor group. It was reported that downregulation of MALAT1 suppressed inflammation by sponging miR-146a in LPS-induced ALI rats [26]. In line with previous research, we found that downregulation of MALAT1 reduced the expression of the pro-inflammatory cytokines TNF-α and IL-1β. The results were also overturned in the MALAT1-shRNA+miR-146a inhibitor group. A previous study has shown that MALAT1 inhibits CD80 transcription by regulating the transcription factor NF-κB binding to the CD80 promoter [38]. Thus, we suspected that NF-κB was associated with the mechanism underlying the protective effects of MALAT1 in ICH rats. Similar to a previous study, we found that MALAT1-shRNA inhibited the NF‑κB pathway in ICH rats. This effect was abrogated in the MALAT1-shRNA+ miR-146a inhibitor group. These results indicate that MALAT1 serves as an important mediator in protecting against ICH by suppressing inflammation and oxidative stress by targeting miR-146a.

## Conclusion

This research revealed that MALAT1 gene silencing could protect against ICH by suppressing inflammation and oxidative stress by targeting miR-146a. Thus, MALAT1 may be a curative target for the clinical treatment of ICH. This might offer new insights into therapeutic approaches for improving ICH treatment.

## Data Availability

The datasets used and/or analyzed during the current study are available from the corresponding author upon reasonable request.

## Research highlights

Long noncoding RNA MALAT1 was upregulated in intracerebral hemorrhage rats;

Downregulation of MALAT1 reduced neurological damage through upregulating miR-146a;

Downregulation of MALAT1 decreased brain edema through upregulating miR-146a;

Downregulation of MALAT1 suppressed inflammation and oxidative stress through upregulating miR-146a.

## References

[1] de Oliveira Manoel Al. Surgery for spontaneous intracerebral hemorrhage. Crit Care. 2020;24:45. 32033578 10.1186/s13054-020-2749-2 PMC7006102

[2] Cai X, Rosand J. The evaluation and management of adult intracerebral hemorrhage. Semin Neurol. 2015;35:638–645. 26595864 10.1055/s-0035-1564687

[3] Hostettler IC, Seiffge DJ, Werring DJ. Intracerebral hemorrhage: an update on diagnosis and treatment. Expert Rev Neurother. 2019;19:679–694. 31188036 10.1080/14737175.2019.1623671

[4] Wang Z, Zhou F, Dou Y, et al. Melatonin alleviates intracerebral hemorrhage-induced secondary brain injury in rats via suppressing apoptosis, inflammation, oxidative stress, DNA damage, and mitochondria injury. Transl Stroke Res. 2018;9:74–91. 28766251 10.1007/s12975-017-0559-x PMC5750335

[5] Zhou J, Tao K, Guo K, et al. Suppression of NDRG2 alleviates brain injury after intracerebral hemorrhage through mitigating astrocyte-drived glutamate neurotoxicity via NF-kappaB/GLT1 signaling. Brain Res. 2020;1729:146600. 31843625 10.1016/j.brainres.2019.146600

[6] Zhu H, Wang Z, Yu J, et al. Role and mechanisms of cytokines in the secondary brain injury after intracerebral hemorrhage. Prog Neurobiol. 2019;178:101610. 30923023 10.1016/j.pneurobio.2019.03.003

[7] Yang Y, Cheng J, Ren H, et al. Tumor FOXP3 represses the expression of long noncoding RNA 7SL. Biochem Biophys Res Commun. 2016;472:432–436. 26718402 10.1016/j.bbrc.2015.12.082

[8] Zhao C, Wang S, Zhao Y, et al. Long noncoding RNA NEAT1 modulates cell proliferation and apoptosis by regulating miR-23a-3p/SMC1A in acute myeloid leukemia. J Cell Physiol. 2019;234:6161–6172. 30246348 10.1002/jcp.27393

[9] Ye F, Gong Y, Chen X, et al. Long noncoding AFAP1-antisense RNA 1 is upregulated and promotes tumorigenesis in gastric cancer. Oncol Lett. 2018;15:7523–7530. 29740481 10.3892/ol.2018.8266 PMC5934723

[10] Lu M, Hu Y, Wu Y, et al. Genome-wide discovery and characterization of long noncoding RNAs in patients with multiple myeloma. BMC Med Genomics. 2019;12:135. 31619233 10.1186/s12920-019-0577-5 PMC6794882

[11] Gu C, Chen C, Wu R, et al. Long noncoding RNA EBF3-AS promotes neuron apoptosis in alzheimer’s disease. DNA Cell Biol. 2018;37:220–226. 29298096 10.1089/dna.2017.4012

[12] Li BG, Wu WJ, Zheng HC, et al. Long noncoding RNA GAS5 silencing inhibits the expression of KCNQ3 by sponging miR-135a-5p to prevent the progression of epilepsy. Kaohsiung J Med Sci. 2019;35:527–534. 31373759 10.1002/kjm2.12102 PMC11900737

[13] Kim JM, Moon J, Yu JS, et al. Altered long noncoding RNA profile after intracerebral hemorrhage. Ann Clin Transl Neurol. 2019;6:2014–2025. 31557399 10.1002/acn3.50894 PMC6801204

[14] Jia J, Zhang M, Li Q, et al. Long noncoding ribonucleic acid NKILA induces the endoplasmic reticulum stress/autophagy pathway and inhibits the nuclear factor-k-gene binding pathway in rats after intracerebral hemorrhage. J Cell Physiol. 2018;233:8839–8849. 29893407 10.1002/jcp.26798

[15] Zhang J, Dong B, Hao J, et al. LncRNA Snhg3 contributes to dysfunction of cerebral microvascular cells in intracerebral hemorrhage rats by activating the TWEAK/Fn14/STAT3 pathway. Life Sci. 2019;237:116929. 31610210 10.1016/j.lfs.2019.116929

[16] Gutschner T, Hammerle M, Eissmann M, et al. The noncoding RNA MALAT1 is a critical regulator of the metastasis phenotype of lung cancer cells. Cancer Res. 2013;73:1180–1189. 23243023 10.1158/0008-5472.CAN-12-2850 PMC3589741

[17] Fang H, Li HF, He MH, et al. Long non-coding RNA MALAT1 sponges microRNA-429 to regulate apoptosis of hippocampal neurons in hypoxic-ischemic brain damage by regulating WNT1. Brain Res Bull. 2019;152:1–10. 31185248 10.1016/j.brainresbull.2019.06.004

[18] Cai LJ, Tu L, Huang XM, et al. LncRNA MALAT1 facilitates inflammasome activation via epigenetic suppression of Nrf2 in Parkinson’s disease. Mol Brain. 2020; 13(1):130. 32972446 10.1186/s13041-020-00656-8 PMC7513532

[19] Yan Y, Song D, Song X, et al. The role of lncRNA MALAT1 in cardiovascular disease. IUBMB Life. 2020 Mar;72(3):334–342. 31856403 10.1002/iub.2210

[20] Strub GM, Perkins JA. MicroRNAs for the pediatric otolaryngologist. Int J Pediatr Otorhi. 2018;112:195–207. 10.1016/j.ijporl.2018.06.043 30055733

[21] Shirazian A, Nguyen DH, and Tran V, et al. Microrna as biomarkers to distinguish intracerebral and subarachnoid hemorrhage pathology. J Neurol Sci. 2019;405:117.

[22] Wang ZY, Yuan BQ, and Fu FL, et al. Hemoglobin enhances miRNA-144 expression and autophagic activation mediated inflammation of microglia via mTOR pathway. Sci Rep-Uk. 2017;7: 11861. 10.1038/s41598-017-12067-2 PMC5605685 28928406

[23] Li LP, Qi C, Liu YY, et al. MicroRNA miR-27b-3p regulate microglial inflammation response and cell apoptosis by inhibiting A20 (TNF-α-induced protein 3). Bioengineered. 2021 Dec;12(2):9902–9913. 34895052 10.1080/21655979.2021.1969195 PMC8810141

[24] Tan Y, Yu LT, Zhang CM, et al. miRNA-146a attenuates inflammation in an in vitro spinal cord injury model via inhibition of TLR4 signaling. Exp Ther Med. 2018;16:3703–3709. 30233729 10.3892/etm.2018.6645 PMC6143872

[25] Qu X, Wang N, Cheng WT, et al. MicroRNA-146a protects against intracerebral hemorrhage by inhibiting inflammation and oxidative stress. Exp Ther Med. 2019;18:3920–3928. 31656540 10.3892/etm.2019.8060 PMC6812313

[26] Dai LL, Zhang GJ, Cheng Z, et al. Knockdown of LncRNA MALAT1 contributes to the suppression of inflammatory responses by up-regulating miR-146a in LPS-induced acute lung injury. Connect Tissue Res. 2018;59:581–592. 29649906 10.1080/03008207.2018.1439480

[27] Zhou XQ, Chang YZ, Zhu LR, et al. LINC00839/miR-144-3p/WTAP (WT1 Associated protein) axis is involved in regulating hepatocellular carcinoma progression. Bioengineered. 2021;12(2):10849–10861. 34634995 10.1080/21655979.2021.1990578 PMC8809969

[28] Yuan B, Shen H, Lin L, et al. MicroRNA367 negatively regulates the inflammatory response of microglia by targeting IRAK4 in intracerebral hemorrhage. J Neuroinflammation. 2015; 12:206. 26552593 10.1186/s12974-015-0424-3 PMC4640168

[29] Chen J, Li Y, Wang L, et al. Therapeutic benefit of intravenous administration of bone marrow stromal cells after cerebral ischemia in rats. Stroke. 2001;32(4):1005–1011. 11283404 10.1161/01.str.32.4.1005

[30] Telford WG. Multiparametric analysis of apoptosis by flow cytometry. Methods Mol Biol. 2018;1678:167–202. 29071681 10.1007/978-1-4939-7346-0_10 PMC8063493

[31] Konstantinou GN. Enzyme-linked immunosorbent assay (ELISA). Meth Mol Bio. 2017;1592:79–94. 10.1007/978-1-4939-6925-8_7 28315213

[32] Livak KJ, Schmittgen TD. Analysis of relative gene expression data using real-time quantitative PCR and the 2(-Delta delta C(T)) method. Methods. 2001;25(4):402–408. 11846609 10.1006/meth.2001.1262

[33] Han D, Yu ZJ, Zhang H, et al. Microenvironment-associated gene HSD11B1 may serve as a prognostic biomarker in clear cell renal cell carcinoma: a study based on TCGA, RT‑qPCR, Western blotting, and immunohistochemistry. Bioengineered. 2021;12:10891–10904. 34845968 10.1080/21655979.2021.1994908 PMC8810109

[34] Wei N, Wei Y, Li B, et al. Baicalein promotes neuronal and behavioral recovery after intracerebral hemorrhage via suppressing apoptosis, oxidative stress and neuroinflammation. Neurochem Res. 2017;42:1345–1353. 28108850 10.1007/s11064-017-2179-y

[35] Sun D, Wang J, Liu X, et al. Dexmedetomidine attenuates endoplasmic reticulum stress-induced apoptosis and improves neuronal function after traumatic brain injury in mice. Brain Res. 2020;1732:146682. 31991122 10.1016/j.brainres.2020.146682

[36] Nong A, Li Q, Huang Z, et al. MicroRNA miR-126 attenuates brain injury in septic rats via NF-κB signaling pathway. Bioengineered. 2021 Dec;12(1):2639–2648. 34115555 10.1080/21655979.2021.1937905 PMC8806573

[37] Zhou XY, Wang ZL, Xu BC, et al. Long non-coding RNA NORAD protects against cerebral ischemia/reperfusion injury induced brain damage, cell apoptosis, oxidative stress and inflammation by regulating miR-30a-5p/YWHAG. Bioengineered. 2021 Dec;12(2):9174–9188. 34709972 10.1080/21655979.2021.1995115 PMC8810080

[38] Juan C, Wang Q, Mao Y, et al. Knockdown of LncRNA MALAT1 contributes to cell apoptosis via regulating NF-kappaB/CD80 axis in neonatal respiratory distress syndrome. Int J Biochem Cell Biol. 2018;104:138–148. 30243953 10.1016/j.biocel.2018.09.009

